# Diagnosis and Genotyping of *Coxiella burnetii* Endocarditis in a Patient with Prosthetic Pulmonary Valve Replacement Using Next-Generation Sequencing of Plasma Microbial Cell-Free DNA

**DOI:** 10.1093/ofid/ofz242

**Published:** 2019-06-01

**Authors:** Maiko Kondo, Sudeb C Dalai, Shivkumar Venkatasubrahmanyam, Nell Eisenberg, Brian D Robinson, Lars F Westblade, Kristen M Marks

**Affiliations:** 1 Division of Infectious Diseases, Department of Medicine, Weill Cornell Medicine, New York, New York; 2 Karius, Inc., Redwood City, California; 3 Division of Infectious Diseases, Stanford University Medical Center, Stanford, California; 4 Department of Medicine, Weill Cornell Medicine, New York, New York; 5 Department of Pathology and Laboratory Medicine, Weill Cornell Medicine, New York, New York

**Keywords:** *Coxiella burnetii*, endocarditis, next-generation sequencing, plasma microbial cell-free DNA, Q fever

## Abstract

Determining the causative etiology of culture-negative endocarditis can be challenging. We performed next-generation sequencing of plasma microbial cell-free DNA to facilitate rapid diagnosis and genotyping of *Coxiella burnetii* in a patient with culture-negative endocarditis of a prosthetic pulmonary valve, enabling early targeted treatment prior to valve replacement surgery.

Culture-negative endocarditis (CNE) comprises approximately 5%–55% of all cases of infective endocarditis and can be diagnostically challenging [1]. When severe valvular disease is present, immediate valve replacement surgery may be necessary prior to definitive microbiologic diagnosis or initiation of appropriate antimicrobial therapy. This increases the risk of re-infecting new prosthetic material. Next-generation sequencing (NGS) of microbial cell-free DNA (mcfDNA) has demonstrated clinical utility in identifying a broad range of pathogens with high sensitivity and short turnaround time [2–4]. We present a case of CNE where NGS of mcfDNA in a patient’s plasma facilitated rapid identification and genotyping of *Coxiella burnetii*, which was subsequently confirmed by serologic testing and detection of *C. burnetii* DNA in the explanted cardiac tissue.

## CASE PRESENTATION

A 29-year-old male with 18 months of intermittent fevers, night sweats, and 6 kg weight loss presented to outpatient cardiology. He had a history of Tetralogy of Fallot with multiple cardiac surgeries, including Blalock shunt placement at age 7 days followed by repair at 3 years of age, homograft pulmonary valve replacement (PVR) in 2006, and bioprosthetic PVR in 2014. Other relevant history included travel to Pakistan, Thailand, Laos, and Myanmar after PVR in 2014, use of a LivaNova 3T Heater-Cooler device during PVR surgery in 2014, and consumption of unpasteurized milk in the Midwestern United States. He denied animal contact.

Vital signs were normal. Physical examination was notable for a holosystolic murmur and hepatosplenomegaly. Initial blood cultures and a fourth-generation HIV screening test were negative. Transesophageal echocardiography demonstrated severely elevated pulmonary artery (PA) pressure and an erratically moving echodensity on the pulmonary valve suggestive of vegetation. The patient was subsequently admitted to the hospital for further management of presumed CNE.

Initial concerns included *Mycobacterium chimaera* prosthetic valve endocarditis given a documented outbreak associated with contaminated LivaNova 3T Heater-Cooler devices [5]. Furthermore, the patient brought a letter from the hospital where the PVR was performed in 2014 warning of possible exposure to *M. chimaera*. Other potential etiologies included *Bartonella henselae*, *Brucella* species, and *C. burnetii*.

**Figure 1.**
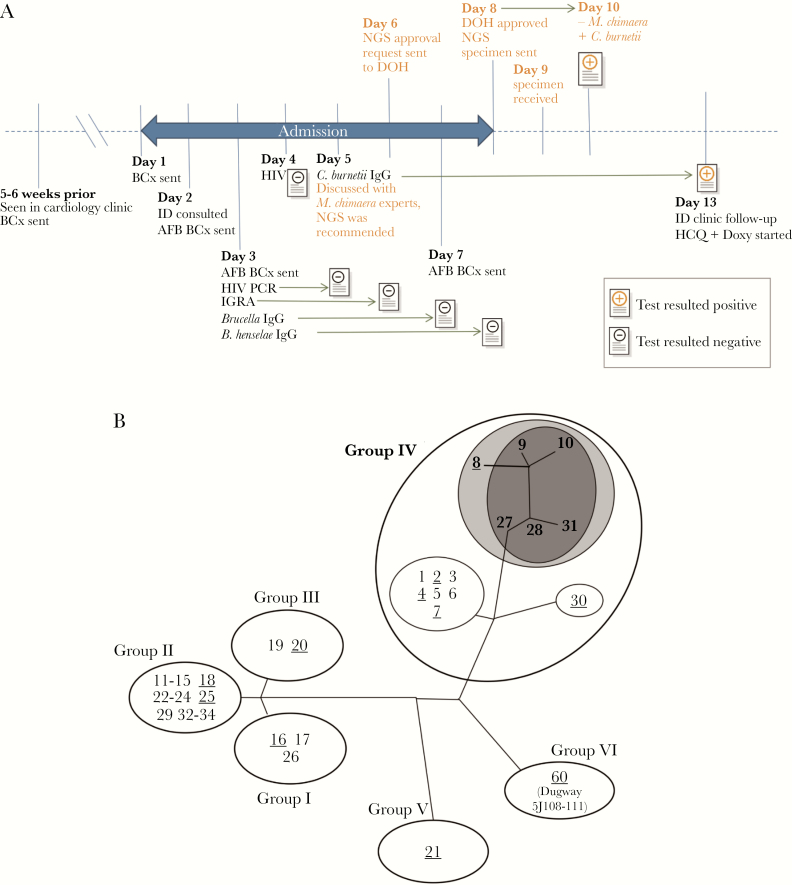
A, Timeline of microbiologic testing. All microbiologic assays (with the exception of plasma mcfDNA NGS) were recommended during the initial infectious diseases consultation (day 2). The day when each assay was sent is shown in the timeline. The position of the result icon correlates with the day each result became available. The timeline of plasma mcfDNA NGS testing is highlighted in orange-colored text. B, Strain typing results in the context of *C. burnetii* phylogeny [7]. Genomic groups and ST numbers are labeled. Of the STs represented by publicly available whole-genome sequences (underlined), the observed *C. burnetii* mcfDNA fragments are best accounted for by ST8 (light gray). Single nucleotide polymorphism analysis of DNA isolated from cardiac tissue placed the offending *C. burnetii* strain closest to STs 9, 10, 27, 28, and 31 (dark gray), for which no whole-genome sequences were publicly available for mcfDNA analysis. All of these closely related strains (STs 8, 9, 10, 27, 28, and 31) belong to Group IV. ST8 is linked with chronic infection in humans (especially endocarditis) and is associated with goats in North America, France, and Spain [7, 8]. In contrast, STs 9, 10, 27, 28, and 31 have been isolated in France, Austria, Kazakhstan [8], and Afghanistan [9]. Abbreviations: AFB, acid-fast bacillus; BCx, blood culture; DOH, New York State Department of Health (the mcfDNA plasma assay requires DOH approval); Doxy, doxycycline; HCQ, hydroxychloroquine; HIV, fourth-generation HIV screening test; HIV PCR, HIV-1 viral load real-time reverse transcription-polymerase chain reaction (RT-PCR); ID, infectious diseases; IGRA, interferon-gamma release assay; mcfDNA, microbial cell-free DNA; NGS, next-generation sequencing of mcfDNA.

Although the patient was clinically stable, his cardiologist had concern for decompensation and sudden cardiac death due to severely elevated PA pressure. To prevent reinfection of new prosthetic material, the cardiologist consulted the infectious diseases service for empiric treatment recommendations before surgery. Upon our recommendation, serologic tests for *B. henselae*, *Brucella* species, *C. burnetii*, and *Legionella pneumophila*, as well as acid-fast bacilli blood cultures were sent. However, empiric therapy for the multiple etiologic agents under consideration, including *M. chimaera*, was not recommended given that it could reduce diagnostic yield from cardiac tissue culture and commit the patient to long-term empiric therapy with toxic antimicrobials (eg, amikacin). Based on advice from a physician with *M. chimaera* expertise, we sent the patient’s plasma for NGS of mcfDNA to facilitate rapid and comprehensive diagnosis including evaluation for *M. chimaera* infection (Figure 1A) [6].

## METHODS

Plasma was collected from the patient at NewYork-Presbyterian Hospital–Weill Cornell Medical Center and sent to a Clinical Laboratory Improvement Amendments–certified and College of American Pathologists–accredited laboratory for NGS (Karius, Inc., Redwood City, CA). Plasma mcfDNA was extracted, and NGS was performed according to previously described methods [2]. Human sequence reads were removed, and the remaining sequences were aligned to a curated database of >1000 pathogens including bacteria, mycobacteria, DNA viruses, fungi, and protozoa. Organisms above a predefined significance threshold were reported [2]. For *C. burnetii* strain typing, alignments to different *Coxiella* strains in the pathogen database were compared by BLAST bit score to determine the most closely related strain to the offending organism. *Coxiella burnetii* serology was performed by ARUP Laboratories (Salt Lake City, UT); IgG Phase I and Phase II titers were obtained by serial dilution.

## RESULTS

Within 48 hours of sample receipt, NGS detected *C. burnetii* mcfDNA. Strain typing using BLAST alignments of *C. burnetii* mcfDNA fragments identified a best match against sequence type (ST) 8 (including strains CbuK_Q154 and MSU Goat Q177) (Supplementary Figure 1), which belongs to genomic Group IV (Figure 1B) as determined by multispacer sequence typing (MST) [7].

Based on the mcfDNA sequencing results, the patient was diagnosed with presumed *C. burnetii* endocarditis. Upon further discussion, the patient recalled witnessing the slaughtering of a goat at close distance in rural Pakistan, a more likely risk factor than the consumption of unpasteurized milk in the United States. Three days later, serologic testing revealed a *C. burnetii* Phase I IgG titer of 1:1 048 576 and a Phase II IgG titer of 1:2 097 152 (Figure 1A). After treatment with hydroxychloroquine (200 mg orally every 8 hours) and doxycycline (100 mg orally twice daily) was initiated, the patient had complete resolution of symptoms after 4 weeks of therapy and ultimately underwent successful PVR and graft exchange.

Hematoxylin and eosin staining of the explanted cardiac tissue demonstrated fibrosis and a chronic inflammatory infiltrate composed of lymphocytes, plasma cells, and macrophages, including scattered giant cells. Cardiac tissue was also sent to the Centers for Disease Control and Prevention (CDC) for further investigation. *Coxiella burnetii* DNA was successfully extracted from formalin-fixed, paraffin-embedded tissue, and strain typing was performed. Genotyping at the CDC using single nucleotide polymorphism (SNP) markers compatible with MST also placed the *C. burnetii* strain in genomic Group IV. SNP typing revealed that the *C. burnetii* strain was closely related to a group of STs including 9, 10, 27, 28, and 31 [8] but was unable to distinguish between these sequence types (Figure 1B).

Six months after the initial diagnosis, serologic titers were repeated to monitor response to antimicrobial and surgical therapy, demonstrating an 8-fold decrease in Phase I and 32-fold decrease in Phase II titers (1:131 072 and 1:65 536, respectively). At the time of this publication, the patient is doing well without recurrence of symptoms and will remain on hydroxychloroquine and doxycycline for at least 24 months [10].

## DISCUSSION

*Coxiella burnetii*, the etiologic agent of Q (Query) fever, is transmitted via zoonosis and is an environmentally ubiquitous, pleomorphic gram-negative coccobacillus that can cause acute systemic illness or chronic infections such as endocarditis [10, 11]. *Coxiella burnetii* does not grow in culture using routine laboratory culture techniques, and thus diagnosis of *C. burnetii* endocarditis typically requires compatible clinical symptoms and serology [1, 10].

We present a case of CNE due to *C. burnetii* that was initially diagnosed by NGS of plasma mcfDNA and subsequently confirmed by serology and polymerase chain reaction amplification of *C. burnetti* DNA from cardiac tissue. Sequencing of mcfDNA facilitated noninvasive, rapid diagnosis of *C. burnetii* endocarditis within 48 hours of sample collection, decreasing the likelihood of *M. chimaera* infection. The significance of the NGS result was further supported by high Phase I and Phase II IgG titers and the patient’s compatible exposure history.

There are several advantages of sequencing plasma mcfDNA. First, it can detect bacterial, fungal, parasitic, and DNA viral pathogens in a single assay, reducing the need for numerous pathogen-specific assays. The turnaround time is rapid (24–48 hours upon receipt of the specimen at the testing laboratory), facilitating early initiation of targeted therapy. The specimen collection is minimally invasive (venipuncture), which may decrease the requirement for invasive procedures such as tissue biopsy. Therefore, plasma NGS can permit earlier diagnosis than nucleic acid testing or culture of surgically obtained cardiac tissue [6]. In addition, strain-level information may be obtained by leveraging the plasma mcfDNA sequence data, which would not be available with serology alone. In this case, genotyping elucidated the nature and location of *C. burnetii* acquisition, which are important for epidemiologic investigations given the organism’s classification as a Category B bioterrorism agent [12]. Strain typing of mcfDNA isolated from our patient was most consistent with ST8 among all publicly available whole-genome sequences. SNP typing of DNA isolated from cardiac tissue at the CDC placed the sample in the closely related ST groups 9, 10, 27, 28, and 31 based on an internal database. Reconciling strain typing information (Figure 1B) and exposure history, the patient likely acquired *Coxiella* infection during international travel, possibly while observing a goat slaughter in rural Pakistan, rather than through consumption of unpasteurized cow’s milk in the United States.

 There are several shortcomings of sequencing plasma mcfDNA: they are generally more expensive than culture-based methods, serology, or nucleic acid amplification tests, and at present do not offer some advantages of culture such as phenotypic antimicrobial susceptibility testing. Finally, it is important to recognize that detection of a wide range of organisms may lead to the identification of atypical organisms of unclear clinical significance, and the false-positive rate of NGS remains uncertain. In such cases, interpretation should be cautious, and involvement of infectious diseases and clinical microbiology specialists is essential.

## CONCLUSIONS

To the best of our knowledge, this is the first report describing the use of NGS of mcfDNA obtained from plasma to diagnose *C. burnetii* CNE. Plasma NGS aided in rapid diagnosis, facilitated exclusion of alternative infectious agents in this complicated case, and allowed inference of strain-level information, supporting further investigations regarding the patient’s acquisition of *C. burnetii*. Future clinical studies are needed to validate mcfDNA sequencing as a useful, robust, and informative diagnostic test in CNE [2].

## Supplementary Data

Supplementary materials are available at *Open Forum Infectious Diseases* online. Consisting of data provided by the authors to benefit the reader, the posted materials are not copyedited and are the sole responsibility of the authors, so questions or comments should be addressed to the corresponding author.

ofz242_suppl_supplementary_figure

ofz242_suppl_supplementary_figure_legend

## Notes

We thank Drs. Gilbert Kersh, Christopher Paddock, Thanhthao Huynh, and Amy Denison from Centers for Disease Control and Prevention (CDC) for their work on immunohistochemistry, genotyping, clinical advice, and review of the manuscript. We also thank Drs. Jonathan Ginns and Ole Vielemeyer from NewYork-Presbyterian Hospital-Weill Cornell Medical Center for their involvement in the patient’s care. We thank Dr. David Hong from Karius, Inc., for his help in facilitating the testing and Dr. Joel Ackelsberg from the New York City Department of Health and Mental Hygiene for his advice on this case. We also thank Dr. Dan Diekema from University of Iowa for his advice on *M. chimaera* diagnosis and Dr. Didier Raoult from Aix-Marseille University for his advice on preoperative *Coxiella* endocarditis treatment duration.

***Financial support. ***This work was supported by the National Institute of Allergy and Infectious Diseases (grant number T32AI007613 to M.K.).

***Potential conflicts of interest. ***M.K., L.F.W., N.E., B.D.R., and K.M.M. have no conflicts of interest. S.D. and S.V. are employees of Karius, Inc. (Redwood City, CA). All authors have submitted the ICMJE Form for Disclosure of Potential Conflicts of Interest. Conflicts that the editors consider relevant to the content of the manuscript have been disclosed.

***Prior presentation. ***IDWeek 2018; October 3–7, 2018; San Francisco, CA; Abstract 154.

***Author contributions. ***M.K., K.M.M., and N.E. cared for the case patient and provided all clinical data. M.K. (with S.D.) wrote the manuscript. S.V. performed NGS analysis, performed genotyping of mcfDNA, and created the figures (with M.K.). B.D.R. contributed pathology slides. L.F.W. and K.M.M. coordinated the study and supervised the overall process. All authors reviewed and revised the manuscript.

## References

[1] Tattevin P, Watt G, Revest M, et al. Update on blood culture-negative endocarditis. Med Mal Infect 2015; 45:1–8. 25480453 10.1016/j.medmal.2014.11.003

[2] Blauwkamp TA, Thair S, Rosen MJ, et al. Analytical and clinical validation of a microbial cell-free DNA sequencing test for infectious disease. Nat Microbiol 2019; 4:663–74. 30742071 10.1038/s41564-018-0349-6

[3] Burnham P, Dadhania D, Heyang M, et al. Urinary cell-free DNA is a versatile analyte for monitoring infections of the urinary tract. Nat Commun 2018; 9:2412 https://www.nature.com/articles/s41467-018-04745-0.pdf 29925834 10.1038/s41467-018-04745-0 PMC6010457

[4] De Vlaminck I, Martin L, Kertesz M, et al. Noninvasive monitoring of infection and rejection after lung transplantation. Proc Natl Acad Sci U S A 2015; 112:13336–41. 26460048 10.1073/pnas.1517494112 PMC4629384

[5] Perkins KM, Lawsin A, Hasan NA, et al. Notes from the field: *Mycobacterium chimaera* contamination of heater-cooler devices used in cardiac surgery - United States. MMWR Morb Mortal Wkly Rep 2016; 65:1117–8. 27740609 10.15585/mmwr.mm6540a6

[6] Nomura J, Rieg G, Bluestone G, et al. Rapid detection of invasive *Mycobacterium chimaera* disease via a novel plasma-based next-generation sequencing test. BMC Infect Dis 2019; 19:371 https://www.ncbi.nlm.nih.gov/pmc/articles/PMC6498503/pdf/12879_2019_Article_4001.pdf 31046692 10.1186/s12879-019-4001-8 PMC6498503

[7] Hornstra HM, Priestley RA, Georgia SM, et al. Rapid typing of *Coxiella burnetii*. PLoS One 2011; 6:e26201. 22073151 10.1371/journal.pone.0026201 PMC3206805

[8] Glazunova O, Roux V, Freylikman O, et al. *Coxiella burnetii* genotyping. Emerg Infect Dis 2005; 11:1211–7. 16102309 10.3201/eid1108.041354 PMC3320512

[9] Kersh GJ, Priestley RA, Hornstra HM, et al. Genotyping and axenic growth of *Coxiella burnetii* isolates found in the United States environment. Vector Borne Zoonotic Dis 2016; 16:588–94. 27304166 10.1089/vbz.2016.1972 PMC5011622

[10] Anderson A, Bijlmer H, Fournier PE, et al. Diagnosis and management of Q fever–United States, 2013: recommendations from CDC and the Q Fever Working Group. MMWR Recomm Rep 2013; 62:1–30. 23535757

[11] Reimer LG Q fever. Clin Microbiol Rev 1993; 6:193–8. 8358703 10.1128/cmr.6.3.193 PMC358281

[12] Madariaga MG, Rezai K, Trenholme GM, Weinstein RA Q fever: a biological weapon in your backyard. Lancet Infect Dis 2003; 3:709–21. 14592601 10.1016/s1473-3099(03)00804-1

