# Supplementary figures and images for: Diagnosis and Genotyping of Coxiella burnetii Endocarditis in a Patient with Prosthetic Pulmonary Valve Replacement Using Next-Generation Sequencing of Plasma Microbial Cell-Free DNA
Source: Open Forum Infect Dis. 2019 Jun 1;6(6):ofz242. doi: 10.1093/ofid/ofz242 (PMC6580995; doi:10.1093/ofid/ofz242)

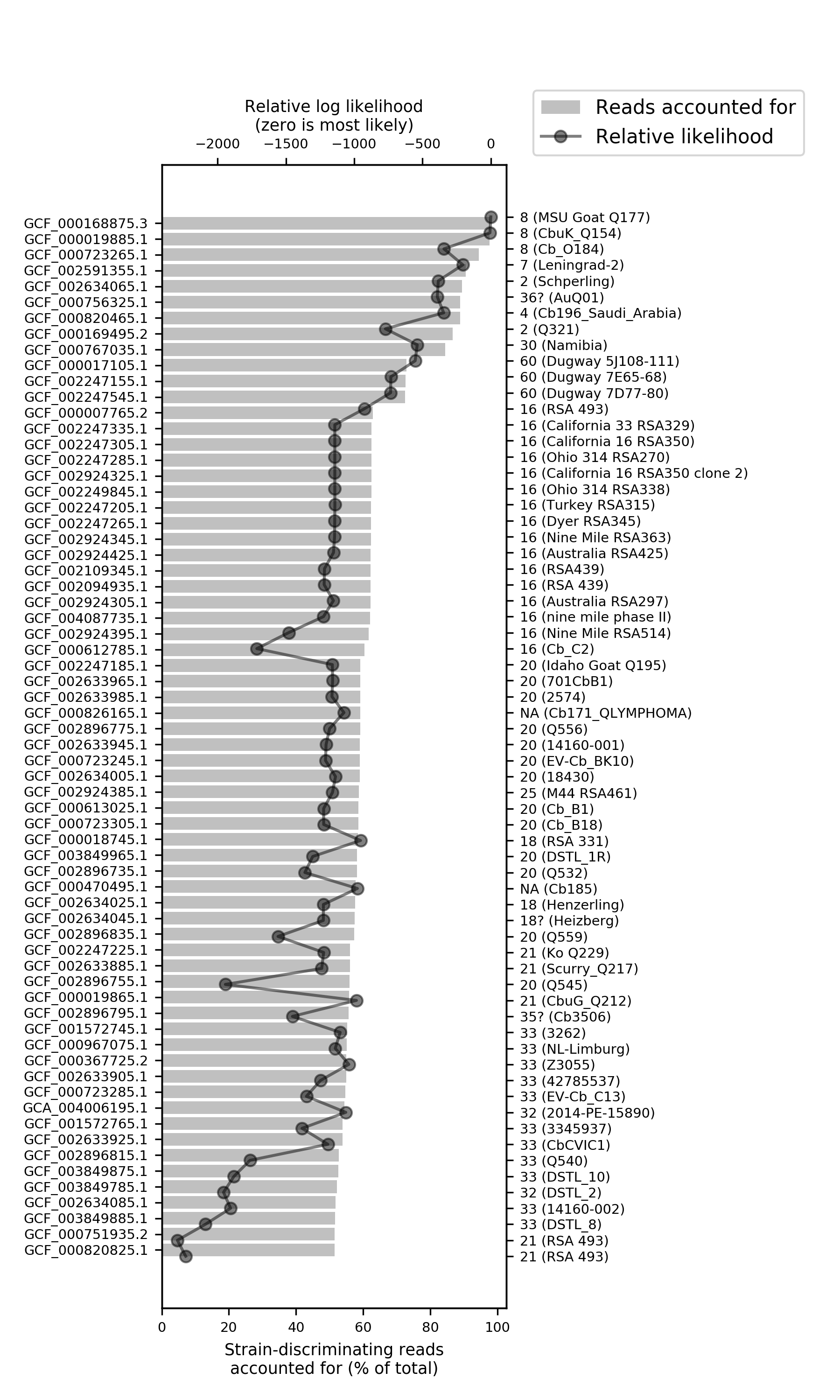

Supplement: ofz242_suppl_supplementary_figure [file ofz242_suppl_supplementary_figure.png]
