# Supplementary material for: Diagnosis and Genotyping of Coxiella burnetii Endocarditis in a Patient with Prosthetic Pulmonary Valve Replacement Using Next-Generation Sequencing of Plasma Microbial Cell-Free DNA
Source: Open Forum Infect Dis. 2019 Jun 1;6(6):ofz242. doi: 10.1093/ofid/ofz242 (PMC6580995; doi:10.1093/ofid/ofz242)
Supplement: ofz242_suppl_supplementary_figure_legend [file ofz242_suppl_supplementary_figure_legend.docx]

**Figure legend**

**Supplemental Figure**: **Strain typing inferred from *C. burnetii* cell-free DNA sequences**. Observed reads that are best accounted for by each of the 66 *C. burnetii* genomes (determined using BLAST bit-score) are shown as light gray bars. The likelihood of each genome under a model that penalizes BLAST mismatches is shown as dark gray points. Assemblies are labelled by NCBI accession on the left, and MST genotype group and strain name on the right.
